# Clinical Neurophysiology, Neuroimaging, and Neuromodulation of Neuropsychiatric Disorders

**DOI:** 10.3390/jpm11111193

**Published:** 2021-11-12

**Authors:** Yoshihiro Noda

**Affiliations:** Department of Neuropsychiatry, Keio University School of Medicine, Tokyo 160-8582, Japan; yoshi-tms@keio.jp; Tel.: +81-3-3353-1211

The goal of this Special Issue is to introduce the cutting-edge research in clinical neurophysiology, neuroimaging, and neuromodulation. We were able to publish 11 original articles and 3 review papers that utilize the latest neuroscience approaches in the abovementioned fields. I would like to introduce these excellent works below.

## 1. EEG Related Research

First, Runnova and Selskii et al. conducted a long-term test in subjects where they maintain attention to sound stimuli, and the authors recorded EEGs and performed wavelet analysis to quantitatively evaluate how EEGs change during the daytime in patients with sleep disorders. In healthy subjects, alpha peak occurred around 9 Hz, but in patients with primary insomnia, the peak was found to increase to around 11 Hz. As for the changes in the dynamics of the alpha rhythm during the process of attention test, there was a significant difference between patients with sleep disorders and healthy controls in the frequency range of 7.5–10.5 Hz, supporting the concept of 24-h hyperarousal in primary insomnia [1].

Second, EEG activity in the brain at gamma frequencies reflects the process of encoding and transmitting information. A 40 Hz auditory steady-state response (40 Hz ASSR) has often been linked to cognitive processing changes in psychiatric disorders; however, the relationship between ASSR and cognitive function remains unclear. Most studies to date have assessed ASSR using a fixed 40 Hz gamma frequency, and less attention has been paid to studies using individual gamma resonance frequencies (IGFs), which are more reflective of individual network characteristics. Parciauskaite et al. focused on processing speeds in various types of cognitive tasks and examined the relationship between 40 Hz and IGF-induced ASSR to determine how IGF is related to certain aspects of cognitive function. The results showed that, when assessing ASSR with 40 Hz and IGF, gamma activity was associated with performance speed in both complex cognitive tasks, i.e., tapping planning and problem solving. However, in the individualized approach with IGF, the observed associations were stronger than with 40 Hz stimulation, and the associations mainly reflected individual differences in higher-order cognitive processing [2].

Third, in treatment-resistant schizophrenia (TRS), abnormal connectivity between the anterior cingulate cortex (ACC) and the default mode network (DMN) has been shown to potentially play an important role in its pathophysiology. Wada et al. aimed to evaluate the connectivity between the ACC and posterior cingulate cortex (PCC), the hub of the DMN, using iCoh, a causal connectivity index. The iCoh values between the PCC and ACC were calculated by sLORETA from 19-channel resting-state EEGs. The results showed that the iCoh ratio in the delta and theta bands with directionality from the left PCC to ACC was lower in the TRS group than in the non-TRS group, suggesting that it may reflect the neurophysiological basis of TRS [3].

Fourth, Shakeel et al. investigated whether forward prediction of time series using an adaptive least mean square (LMS)-based autoregressive (AR) model could be implemented in a real-time closed-loop system by applying state-dependent triggers to EEG alpha oscillation peaks and troughs. During the resting state and a visual task, the proposed method successfully triggered at a specific phase of the EEG oscillations for all subjects. These results indicate that the LMS-based AR model is an adaptive approach that can be implemented in real-time closed-loop systems for specific phases of alpha oscillations, and can be used as an alternative to traditional or machine learning approaches with a lower computational load [4].

Fifth, EEG can be a powerful tool to assess the effects of substance use disorders (SUD) on cognitive functioning. Specifically, modulated gamma activity may be an indicator of the pathophysiology of SUDs. Ramlakhan et al. systematically reviewed the effects of alcohol, tobacco, cannabis, cocaine, and amphetamines on gamma activity of acute and chronic exposure and withdrawal states in preclinical and clinical populations. The results showed that all of the abovementioned substances were associated with modulation of gamma activity, in both preclinical and clinical populations, regardless of the state. However, the effect of alcohol on gamma activity is complex, indicating that it may affect differently from other substances. Thus, the modulation of gamma activity is involved in the pathophysiology of SUD, suggesting the possibility of novel therapies targeting this neurophysiological substrate [5].

## 2. Combined TMS and EMG/EEG Research

First, Noda administered 1 Hz-rTMS to the right DLPFC in healthy subjects and examined its acute neurophysiological effects using combined TMS-EEG. A 1 Hz-rTMS was administered at rest and during a verbal task, and TMS-related power and TMS-related coherence at the F4 and F3 electrode sites were calculated. The results showed that TMS-related power at rest was significantly increased by 1 Hz-rTMS at the stimulation site in alpha, beta, and gamma bands, and TMS-related power during verbal task was significantly increased in alpha and beta bands. On the other hand, TMS-related coherence was significantly increased by 1 Hz-rTMS to the right DLPFC in the resting state in alpha and beta bands, but not in gamma band. Therefore, 1 Hz-rTMS to the right DLPFC may rapidly neuromodulate EEG activity, which may be relevant to the treatment mechanism of depression [6].

Second, Noda et al. compared the amplitudes and latencies of each TMS-evoked potential (TEP) component evoked by single-pulse TMS (spTMS) to the left M1 and DLPFC between groups of healthy young subjects, older subjects, and schizophrenia patients to clarify the spatiotemporal characteristics of each group. Compared with healthy young, the amplitudes of N45 and P180 were decreased by M1-spTMS in the elderly and P180 in patients. On the other hand, N45 was reduced by DLPFC-spTMS in the elderly. Furthermore, in the elderly, P60 was delayed after M1-spTMS and N45-P60 was delayed in the right median after left DLPFC-spTMS, whereas in patients, N45-P60 was delayed in the right central area after DLPFC-spTMS. Our results suggest that the inhibitory and excitatory mechanisms, as indexed by TEP, may be altered in the elderly and in schizophrenia patients [7].

Third, Fisicaro et al. performed single-pulse TMS on patients with Parkinson’s disease (PD) and progressive supranuclear palsy (PSP) to compare neurophysiological characteristics, including motor evoked potentials (MEPs) and cortical suppression periods (CSPs), and to determine whether these differences were related to cognitive function. The results showed that the amplitude of MEPs was higher in the patient groups than in healthy controls, and there was no difference between PD and PSP groups; CSPs were longer in both M1 in patient groups than in healthy controls, but were similar between patient groups. Furthermore, RMT was positively correlated with frontal lobe function in the PSP group, indicating that RMT may be an indicator reflecting cognitive decline in PSP [8].

Fourth, the glutamatergic hypothesis and excitatory/inhibitory (E/I) imbalance hypothesis have been proposed as new hypotheses for the pathophysiology of schizophrenia (SCZ). TMS-EEG is an attractive tool to study the neurophysiology of the human cortex. Li et al. systematically reviewed TMS-EEG studies that investigated cortical functions in SCZ to examine whether TMS-EEG is a suitable modality to account for the above hypotheses. The results suggested that TMS-EEG for SCZ patients showed E/I deficits in the prefrontal cortex, which may be related to cognitive impairment and clinical severity. Furthermore, TMS-induced gamma activity of the prefrontal cortex was associated with positive symptoms, while EEG activity in theta and delta bands was associated with negative symptoms. Thus, TMS-EEG neurophysiology may provide a physiological basis for the establishment of biomarkers for better diagnosis of SCZ and the development of novel treatment strategies [9].

## 3. Neuromodulation Intervention Research

First, rTMS for tinnitus has the advantage that effects can be assessed immediately after intervention, making it an excellent model for testing individualization of rTMS. In this context, Schoisswohl et al. aimed to investigate the reliability of test-retest in modifying tinnitus loudness and oscillatory brain activity with brief rTMS and to examine the feasibility of individualizing rTMS for tinnitus. Patients with tinnitus received brief active and sham stimulation, and EEG was measured before and after each protocol, as well as tinnitus loudness assessment. As a result, they could identify individual rTMS protocols characterized by reliable tinnitus loudness changes (55% of behavioral responders), increased alpha power with parieto-occipital dominance (91% of alpha responders), and decreased gamma power with frontal dominance (100% of gamma responders), respectively. This study demonstrates the potential for individualization using neurophysiological markers rather than behavioral responses, suggesting that individualization of stimulation protocols is possible despite the lack of group-level reliability [10].

Second, Kashyap et al. introduced the dose-target determination index (DTDI), the ratio of the average current density in the target ROI to the ROI with the maximum value (peak region), to quantify the focality of transcranial direct current stimulation (tDCS) and examined the dose-focality relationship in three populations: young adults, middle-aged adults, and older adults. Frontal montage was performed with the target ROI set to the left middle frontal gyrus. As a result, they found that nonlinearity was dominant and focality decreased in the older adults, and this decrease was stronger in males. Therefore, it is clear that increasing the current level can increase the focality of the stimulus in this population, and that DTDI can provide information on which tDCS current level optimizes the focality of the stimulus [11].

Third, vagal nerve stimulation (VNS) has been shown to be effective in the treatment of depression, but until recently VNS devices have required surgical implantation, which has limited their widespread use. However, a novel non-invasive VNS (nVNS) device that can externally stimulate the vagal nerve was developed, raising hopes for its clinical application in stress-related psychiatric disorders. Bremner et al. systematically reviewed the effects of nVNS on physiological functions of patients with stress-related psychiatric disorders in the perspectives of brain imaging, blood inflammation biomarkers, and wearable sensing devices. The results suggest that nVNS favorably affects central brain regions involved in the regulation of autonomic tone, cardiovascular function, inflammatory response, and emotion, and that dysregulation of these circuits and systems may constitute the pathological basis for stress-related psychiatric disorders such as post-traumatic stress disorder (PTSD) and depression. The application of nVNS to stress-related psychiatric disorders may lead to the prevention and treatment of these disorders [12].

Fourth, Noda et al. investigated the effects of gamma frequency violet light (VL) stimulation on human EEG in healthy subjects by comparing it with white light (WL) under the same conditions. Comparing the power spectral density (PSD) of 40 Hz-VL EEG with that of 40 Hz-WL EEG, 40 Hz-VL had significantly lower enhancement of delta and theta bands than 40 Hz-WL. In the occipital region, the negative peak of VEP of 40 Hz-VL was smaller than that of 40 Hz-WL. Furthermore, 40 Hz-VL showed increased alpha-phase and gamma-amplitude coupling compared to the baseline EEG at the F5 electrode site during stimulation and at the C4 electrode site immediately after stimulation. Therefore, 40 Hz-VL stimulation could induce a unique photobiological neuromodulation of human EEG activity [13].

## 4. MRI Neuroimaging Research

Lastly, Sato et al. measured the volumes of the amygdala, hippocampus, Heschl’s gyrus, and temporal lobe in neuroanatomically defined regions of interest (ROIs) using high-resolution MRI in healthy subjects, patients with schizophrenia, and patients with bipolar disorder. The results showed that there was a difference in the right hippocampal volume between the groups (healthy control group > bipolar group > schizophrenia group), with the schizophrenia group showing the smallest value. The study suggested that the right hippocampal volume could be a biomarker for differentiating between schizophrenia and bipolar disorder [14].

As introduced above, I believe that translational and clinical research using state-of-the-art technologies in the field of neuropsychiatric disorders will continue to flourish and contribute to the health and well-being of people. Last but not least, we are currently working on a second part to this Special Issue (https://www.mdpi.com/journal/jpm/special_issues/Neuromodulation_Neuropsychiatric_Series_II) and are looking forward to receiving additional excellent submissions.

## Data Availability

Not applicable.

## References

[1] Runnova A., Selskii A., Kiselev A., Shamionov R., Parsamyan R., Zhuravlev M. (2021). Changes in EEG Alpha Activity during Attention Control in Patients: Association with Sleep Disorders. J. Pers. Med..

[2] Parciauskaite V., Pipinis E., Voicikas A., Bjekic J., Potapovas M., Jurkuvenas V., Griskova-Bulanova I. (2021). Individual Resonant Frequencies at Low-Gamma Range and Cognitive Processing Speed. J. Pers. Med..

[3] Wada M., Nakajima S., Tarumi R., Masuda F., Miyazaki T., Tsugawa S., Ogyu K., Honda S., Matsushita K., Kikuchi Y. (2020). Resting-State Isolated Effective Connectivity of the Cingulate Cortex as a Neurophysiological Biomarker in Patients with Severe Treatment-Resistant Schizophrenia. J. Pers. Med..

[4] Shakeel A., Onojima T., Tanaka T., Kitajo K. (2021). Real-Time Implementation of EEG Oscillatory Phase-Informed Visual Stimulation Using a Least Mean Square-Based AR Model. J. Pers. Med..

[5] Ramlakhan J.U., Ma M., Zomorrodi R., Blumberger D.M., Noda Y., Barr M.S. (2021). The Role of Gamma Oscillations in the Pathophysiology of Substance Use Disorders. J. Pers. Med..

[6] Noda Y. (2021). Potential Neurophysiological Mechanisms of 1Hz-TMS to the Right Prefrontal Cortex for Depression: An Exploratory TMS-EEG Study in Healthy Participants. J. Pers. Med..

[7] Noda Y., Barr M.S., Zomorrodi R., Cash R.F.H., Lioumis P., Chen R., Daskalakis Z.J., Blumberger D.M. (2021). Single-Pulse Transcranial Magnetic Stimulation-Evoked Potential Amplitudes and Latencies in the Motor and Dorsolateral Prefrontal Cortex among Young, Older Healthy Participants, and Schizophrenia Patients. J. Pers. Med..

[8] Fisicaro F., Lanza G., Cantone M., Ferri R., Pennisi G., Nicoletti A., Zappia M., Bella R., Pennisi M. (2020). Clinical and Electrophysiological Hints to TMS in De Novo Patients with Parkinson’s Disease and Progressive Supranuclear Palsy. J. Pers. Med..

[9] Li X., Honda S., Nakajima S., Wada M., Yoshida K., Daskalakis Z.J., Mimura M., Noda Y. (2021). TMS-EEG Research to Elucidate the Pathophysiological Neural Bases in Patients with Schizophrenia: A Systematic Review. J. Pers. Med..

[10] Schoisswohl S., Langguth B., Hebel T., Abdelnaim M.A., Volberg G., Schecklmann M. (2021). Heading for Personalized rTMS in Tinnitus: Reliability of Individualized Stimulation Protocols in Behavioral and Electrophysiological Responses. J. Pers. Med..

[11] Kashyap R., Bhattacharjee S., Arumugam R., Bharath R.D., Udupa K., Oishi K., Desmond J.E., Chen S.H.A., Guan C. (2021). Focality-Oriented Selection of Current Dose for Transcranial Direct Current Stimulation. J. Pers. Med..

[12] Bremner J.D., Gurel N.Z., Wittbrodt M.T., Shandhi M.H., Rapaport M.H., Nye J.A., Pearce B.D., Vaccarino V., Shah A.J., Park J. (2020). Application of Noninvasive Vagal Nerve Stimulation to Stress-Related Psychiatric Disorders. J. Pers. Med..

[13] Noda Y., Takano M., Hayano M., Li X., Wada M., Nakajima S., Mimura M., Kondo S., Tsubota K. (2021). Photobiological Neuromodulation of Resting-State EEG and Steady-State Visual-Evoked Potentials by 40 Hz Violet Light Optical Stimulation in Healthy Individuals. J. Pers. Med..

[14] Sato J., Hirano Y., Hirakawa N., Takahashi J., Oribe N., Kuga H., Nakamura I., Hirano S., Ueno T., Togao O. (2021). Lower Hippocampal Volume in Patients with Schizophrenia and Bipolar Disorder: A Quantitative MRI Study. J. Pers. Med..

